# Structural–Functional Features of the Thyrotropin Receptor: A Class A G-Protein-Coupled Receptor at Work

**DOI:** 10.3389/fendo.2017.00086

**Published:** 2017-04-24

**Authors:** Gunnar Kleinau, Catherine L. Worth, Annika Kreuchwig, Heike Biebermann, Patrick Marcinkowski, Patrick Scheerer, Gerd Krause

**Affiliations:** ^1^Institute of Experimental Pediatric Endocrinology, Charité-Universitätsmedizin, Berlin, Germany; ^2^Group Protein X-Ray Crystallography and Signal Transduction, Institute of Medical Physics and Biophysics, Charité-Universitätsmedizin, Berlin, Germany; ^3^Leibniz-Institut für Molekulare Pharmakologie (FMP), Berlin, Germany

**Keywords:** thyroid-stimulating hormone receptor structure, signal transduction, homology models, glycoprotein hormone receptors, arrestin interaction, G-protein interaction, structure–function relationships, oligomers

## Abstract

The thyroid-stimulating hormone receptor (TSHR) is a member of the glycoprotein hormone receptors, a sub-group of class A G-protein-coupled receptors (GPCRs). TSHR and its endogenous ligand thyrotropin (TSH) are of essential importance for growth and function of the thyroid gland and proper function of the TSH/TSHR system is pivotal for production and release of thyroid hormones. This receptor is also important with respect to pathophysiology, such as autoimmune (including ophthalmopathy) or non-autoimmune thyroid dysfunctions and cancer development. Pharmacological interventions directly targeting the TSHR should provide benefits to disease treatment compared to currently available therapies of dysfunctions associated with the TSHR or the thyroid gland. Upon TSHR activation, the molecular events conveying conformational changes from the extra- to the intracellular side of the cell across the membrane comprise reception, conversion, and amplification of the signal. These steps are highly dependent on structural features of this receptor and its intermolecular interaction partners, e.g., TSH, antibodies, small molecules, G-proteins, or arrestin. For better understanding of signal transduction, pathogenic mechanisms such as autoantibody action and mutational modifications or for developing new pharmacological strategies, it is essential to combine available structural data with functional information to generate homology models of the entire receptor. Although so far these insights are fragmental, in the past few decades essential contributions have been made to investigate in-depth the involved determinants, such as by structure determination *via* X-ray crystallography. This review summarizes available knowledge (as of December 2016) concerning the TSHR protein structure, associated functional aspects, and based on these insights we suggest several receptor complex models. Moreover, distinct TSHR properties will be highlighted in comparison to other class A GPCRs to understand the molecular activation mechanisms of this receptor comprehensively. Finally, limitations of current knowledge and lack of information are discussed highlighting the need for intensified efforts toward TSHR structure elucidation.

## Introduction

The thyroid-stimulating hormone (TSH) or thyrotropin (1) receptor (TSHR) (2–6) is a member of the class A G-protein-coupled receptors (GPCRs) (7). Evolutionary close relatives are the two receptors for the gonadotrophic hormones: follitropin (FSH) (8) and lutropin (LH)/choriogonadotropin (CG) (9). The follicle-stimulating hormone receptor (FSHR) and the LHCGR together with the TSHR constitute the sub-family of glycoprotein hormone receptors (GPHRs) (10). The TSHR is essential for thyroid growth and function (11–13) and activates different G-protein subtypes (14–17) and signaling pathways (18–20), whereby Gs- and Gq-induced signaling are probably of highest importance (13, 21–24). TSH and its receptor are required for thyroid hormone synthesis and release in the thyroid gland (25). Dysfunctions of the TSHR are the underlying cause of various gain- or loss-of-function phenotypes associated with thyroid malfunction [reviewed in Ref. (26)]. It has been suggested that the TSHR is involved in the development and mechanisms of ophthalmopathy (16, 27–31).

For decades, the TSHR and associated molecular mechanisms, such as ligand binding (32, 33), cell-surface expression, or induced signaling cascades, were studied with the purpose to not only understand the different steps in signal transduction, their regulation, and specificity but also to receive insights into the related physiological aspects (13, 20, 34–38) or to develop tools for pharmacological treatment (39, 40). Consequently, a huge amount of specific data and information from genetic approaches (site-directed modifications), pathogenic conditions, protein structure studies, biochemical and biophysical analyses are available [see also the *information resource* of Sequence Structure Function Analysis for *GPHR* at http://www.ssfa-gphr.de (41–44) which contains >1,500 pathogenic and site-directed mutations; comparison of functional data enabled due to normalization as percentage of wild type (WT)].

This raises the following questions, what do we currently know about the complex scenario of signal transduction by the TSHR and what is currently far from our understanding? To answer these questions, here we summarize and discuss the current knowledge about the TSHR with a specific emphasis on structural aspects of receptor activation. This comprises the TSHR structure itself, complexes between this receptor and interacting proteins, and also the transition between different conformations related to different functional processes. For these purposes, the available—albeit fragmental—structural information for the TSHR and its interacting proteins will first be described followed by an assembling of this knowledge into homology models of the entire receptor highlighting the structural and functional specificities in relation to the signal transduction processes.

**Figure 1:**
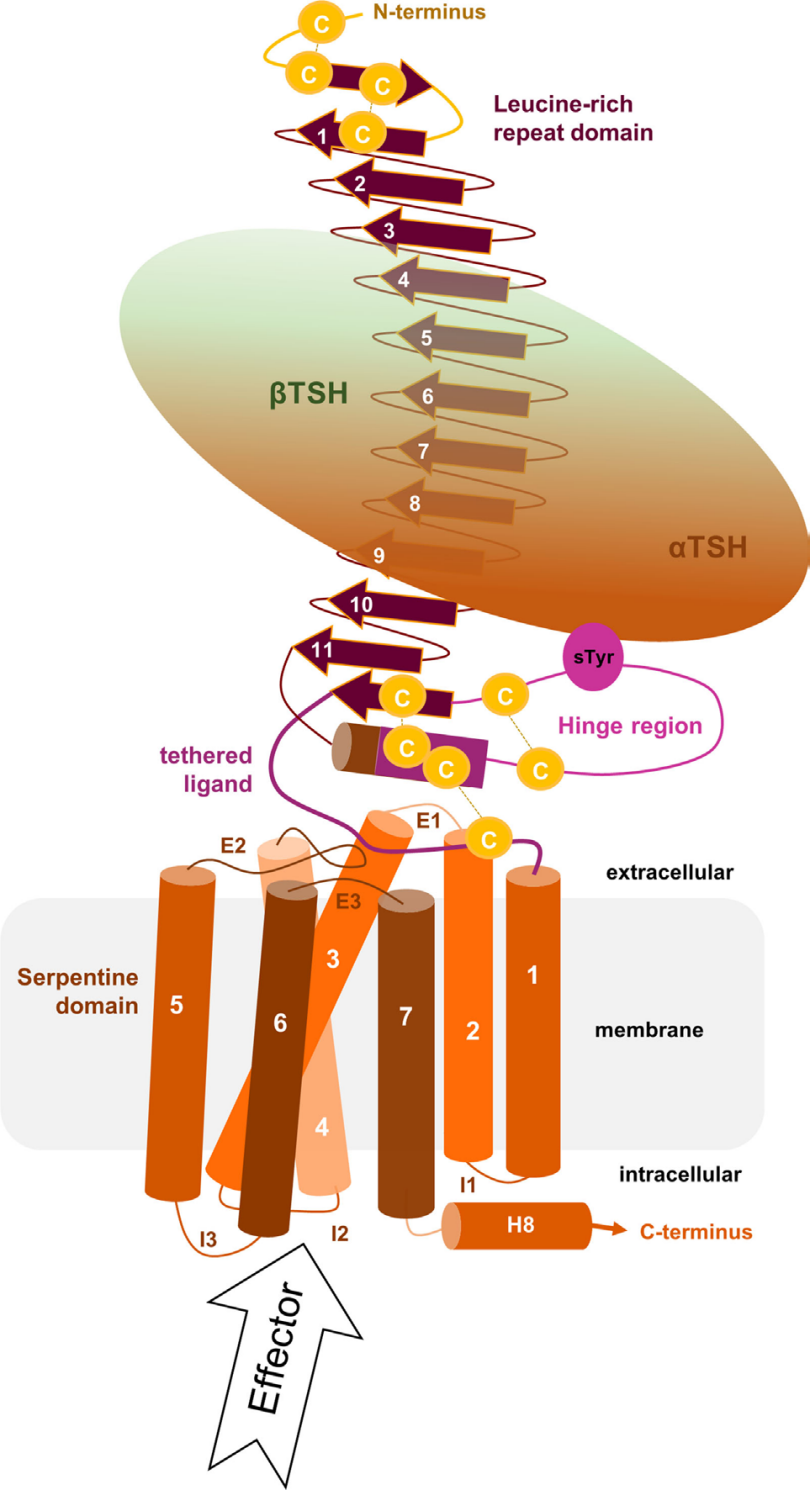
**Scheme of the putative overall thyroid-stimulating hormone receptor (TSHR) protein structure**. This scheme shows the overall structure and domain assembly of the TSHR. Significant features are highlighted, e.g., the sulfated tyrosine in the hinge region that is involved in hormone binding. The leucine-rich repeat domain (LRRD) together with the hinge region constituting the extracellular receptor part. The seven transmembrane helices and their connecting loops arrange the serpentine domain, which spans the membrane from the extra- to the intracellular side. A tethered ligand located between the extracellular loops has been proven and is composed of amino acids from both C-terminal ends of the LRRD and the hinge region.

For understanding of “signal transduction” and related details described in the following sections, it is essential to keep in mind that the 3-dimensional TSHR structure is constituted by interplaying domains (Figure 1) located in different cellular environments. This fact is due to the principal molecular function of GPCRs as hubs to transduce signals. The “signal” is induced by ligand binding at the extracellular site and transmitted *via* structural rearrangements in the transmembrane-spanning receptor region [serpentine domain (SD) comprised transmembrane helices including their connecting loops] toward intracellular effectors. A receptor like the TSHR therefore not only receives a signal but it is also a trigger, catalyzer, and regulator for specific physical or biophysical information. Moreover, the communication inside the protein is regulated by several specific amino acids or groups of amino acids at diverse structural parts that are responsible, for instance, for intermolecular contacts (e.g., for ligand binding) or intramolecular interactions (e.g., for maintenance of a specific conformation). In consequence, each part of the receptor has individual functional priorities that are interrelated with highly adapted structural features. The entire process of signal transduction is a sequence of concerted events that are disturbed under pathogenic conditions and must be circumvented by pharmacological interventions (45–52).

## Available Structural Information

### The Extracellular Leucine-Rich Repeat Domain (LRRD) and the Hinge Region

The extracellular LRRD and hinge region of the TSHR constitute the N-terminal extracellular receptor part (Figure 1), which is remarkably large (around 400 amino acids) compared to other class A GPCRs (10, 53). TSH and antibodies (activating, neutral, and blocking antibodies) interact with the receptor in this region [e.g., Ref. (54–58)]. The LRRD and the hinge region contain six asparagine-linked glycosylation sites (N-Xaa-S/T) that were already investigated intensively (59–62), and it was suggested that glycosylation of at least four sites appears necessary for expression of the functional TSHR (59).

**Figure 2:**
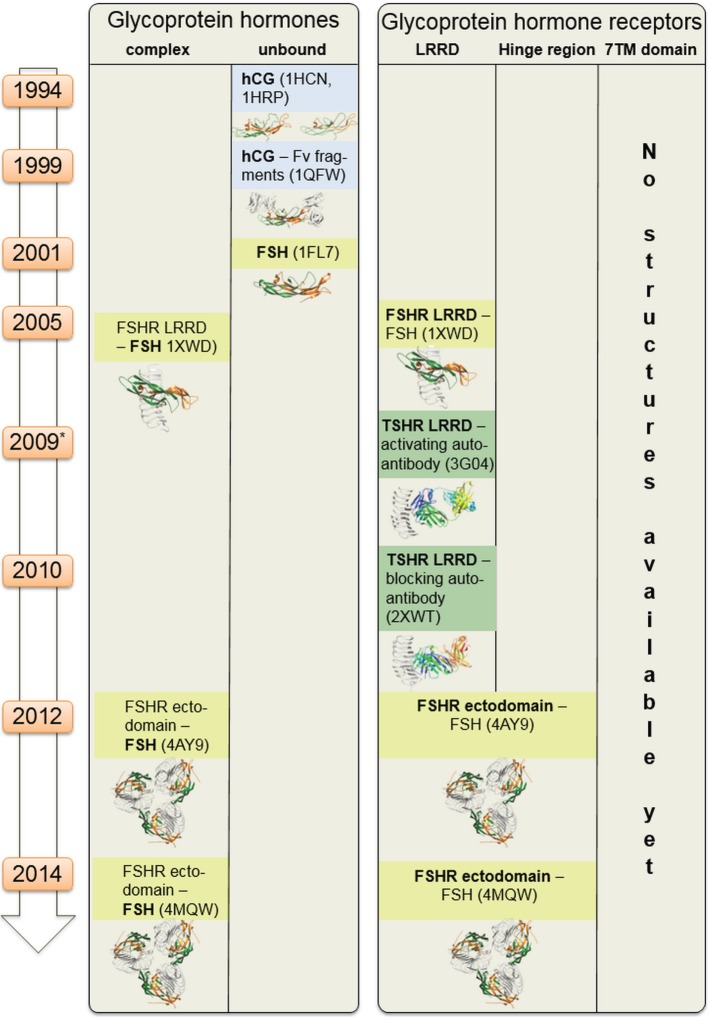
**Available structural information for glycoprotein hormone receptors (GPHRs) and GPHs**. This scheme summarizes structural information that is available for the GPHRs and GPHs. Since 1994 starting with the first crystal structure of human choriogonadotropin, few further endogenous ligand structures (such as from follitropin or thyroid-stimulating hormone receptor autoantibodies—in complexes or unbound) were solved. Based on the high amino acid sequence similarity, each of these structures can also serve as structural templates for models of receptors and hormones where no structural information is available so far. Moreover, these structural data can be assembled into larger complexes (see Figures 6–8).

**Figure 3:**
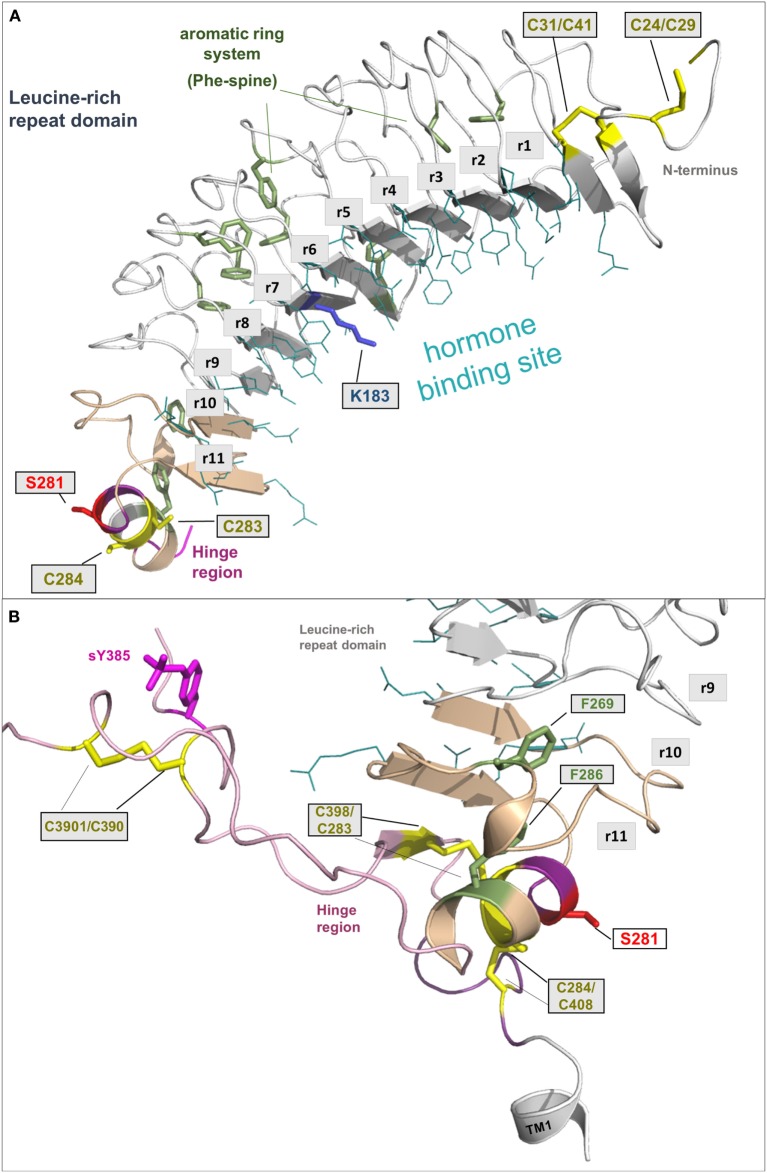
**A full-length model of the thyroid-stimulating hormone receptor (TSHR) leucine-rich repeat domain and a fragmental model of the hinge region**. **(A)** The LRRD of the TSHR is the main binding site for hormones and autoantibodies. They interact with amino acids in the concave site of this domain, which is arranged as a beta-sheet. Hydrophobic amino acid side chains are located mainly in the inner core of the domain, thus aromatic interactions are of high importance. Although the so far solved TSHR LRRD structures are constituted by a maximum of 9 repeats (56, 57), it was suggested (53) that this domain is actually constituted by 11 repeats (r1–r11)—as also presented here in this model (designed by a chimeric model-approach, LRRD model comprises amino acids 24–288). In contrast to other known LRRD structures with similarity to the glycoprotein hormone receptor (GPHR) LRRDs (66–68), the backbone on the convex side of this domain shows only one short helical structure namely in repeat 11. The cysteines at positions 283 and 284 are known to interact with two cysteines at the C-terminal hinge region **(B)**. Furthermore, mutations of serine 281 were identified as pathogenic (70, 71) and causing a gain of function by constitutive receptor activation. Of note, lysine 183 in repeat 7 (blue stick) was identified to be highly responsible for ligand specificity. The Lys183Arg substitution leads to a hypersensitivity for choriogonadotropin (74, 75). **(B)** This fragmental TSHR hinge region model (lilac-purple, amino acids 289–304 and 382–409) is adapted according to the solved follicle-stimulating hormone receptor (FSHR) ectodomain (ECD)/FSH complex structure (65) and contains several amino acids of high structural and functional importance. The cysteine 398 is located in a small beta-strand that is arranged parallel to the last beta-strand 11 of the LRRD. The two essential disulfide bridges Cys283/Cys398 and Cys284/Cys408 are shown. A third extracellular disulfide bridge between Cys301 and Cys390 stabilizes the interplay between the N- and C-terminus. Moreover, the recent FSHR ECD crystal structure bound with follitropin provided details for the first time on the second hormone-binding site of GPHRs around a conserved sulfated tyrosine (in TSHR sTyr385). This tyrosine binds into a pocket between the hormone subunits and contributes to ligand-binding properties (76).

The LRRD comprises repeats of specific amino acid sequences between 20 and 30 residues in length [for a detailed description of GPHR LRRD repeats, see Ref. (63)] known from available TSHR and FSHR crystal structures (56, 57, 64, 65) (Figure 2). The LRRD has a scythe blade-like shape with a slight twist from the N- toward the C-terminus. Hydrophobic amino acid side chains stabilize the inner core of the LRRD and aromatic interactions specifically are of high importance to maintain the backbone of the assembled repeats (Figure 3). Although the so far solved TSHR LRRD crystal structures showed a maximum of nine repeats (56, 57), based on homology modeling combined with mutagenesis studies (53), it was suggested that this domain is actually composed of 11 repeats (r1–r11 in Figure 3)—which was confirmed afterward by the recently solved FSHR LRRD structure (65). Interestingly, in contrast to other LRRDs with a similar fold (66–68), only the last C-terminal repeat of the GPHR LRRD is characterized by a short helix motif. Located in this helix are two cysteines at positions 283 and 284 that are known to interact with two cysteines at the C-terminal hinge region (65, 69). These disulfide bridges are important for adjusting both extracellular parts to each other and simultaneously anchoring the entire extracellular region close to the SD (Figure 3B). Moreover, gain-of-function mutations at position serine 281 leading to constitutive receptor activation were identified in patients (70, 71). This amino acid is also located in the helical part of the LRRD C-terminus and is crucial for activation (69, 70, 72, 73).

In the GPHR subfamily, the hinge region structurally links the LRRD with the SD (77). Unfortunately, little is known about the entire structure of the TSHR hinge region for several reasons. First of all, the TSHR hinge region is most likely not a self-folding domain (53). It might be that only parts of this region are specifically folded, or that interacting receptor fragments and/or the bound ligand are necessary to stabilize the hinge region in a specific conformation.

Related to this is the fact that the TSHR can be enzymatically cleaved at two sites in the hinge region (78, 79), which is also a prerequisite for shedding (78, 80–84) of the disulfide bridges located between the LRRD and the hinge region or inside the hinge region (Figures 1 and 3B). Shedding and cleavage in combination finally releases the so-called “receptor-subunit A” (constituted by the LRRD and parts of the hinge region) from the “receptor-subunit B” (C-terminal part of the N-terminus together with the SD) and cleavage plus shedding are unique to the TSHR in the group of GPHRs. This separation is likely related to the pathogenic occurrence of autoimmune antibodies against the TSHR (20, 34, 79, 85, 86). The cleaved peptide is termed “C-peptide” (approximately 50 amino acids in length), and it is still under debate how this process is related to physiological functions, signaling regulation, or pathogenic conditions (79, 84, 87–89). In any case, it is completely unknown how the C-peptide is folded or contributes to inter- and intramolecular interactions. This question remains important for understanding differences among the GPHRs.

From the crystal structure complex of FSHR ectodomain (ECD)/FSH only fragments of the hinge region are known, with a portion in the middle of the hinge region being unresolved (65). This missing part corresponds to TSHR residues 305–380. The entire TSHR hinge region is predicted to span positions 289–409 (53). However, the solved FSHR ECD crystal structure and derived models for the ECD TSHR (90, 91) highlight that the N- and C-terminus of the hinge region are essential for receptor functions like TSH binding and signal transduction. In detail, a third extracellular disulfide bridge between Cys301 and Cys390 [which is not conserved in GPHRs in general, reviewed in Ref. (9)] constrains the close interplay between the N- and C-terminus of the hinge region (Figure 3B). Cysteine 398 is located in a small beta-strand that is arranged parallel to the last beta-strand of the LRRD. This feature stabilizes the LRRD/hinge region complex, which may explain together with the two essential disulfide bridges Cys283/Cys398 and Cys284/Cys408 why this part was also solved in the FSHR crystal structure (65).

Moreover, the FSHR ECD crystal structure bound with FSH provided for the first time details of the second hormone-binding site of GPHRs around a conserved sulfated tyrosine (sTyr) (functionally corresponds to sTyr385 in TSHR). This tyrosine binds into a pocket between the hormone subunits and strongly contributes to hormone-binding properties (76), although small differences among the GPHRs were observed (92, 93). Generally, the hinge region of GPHRs is the least conserved receptor part (10, 63) and is therefore responsible for several differences concerning associated functions like hormone binding or induction of signaling pathways (94, 95).

### The Membrane-Spanning SD

Currently, no structural information for the SD, comprising the seven membrane-spanning helices and respective connecting loops, has been experimentally determined yet for the TSHR or other GPHRs (Figure 2). This precludes detailed insights being made about amino acid interactions (at the atom level) and also the arrangement of the domains (SD, LRRD, and hinge region) or complexes to each other. However, it can be assumed that the TSHR has the same general assembly of the transmembrane helices as observed for all class A GPCRs because they share a common structural organization (96–99). Thus, experimentally determined structures of other GPCRs can be used as a proxy to generate TSHR models by using homology modeling techniques (100–103). This has been done several times in the past for different purposes [e.g., Ref. (45, 90, 104–107)]. These models were helpful for elucidating mechanisms of pathogenic mutations (26, 108, 109), allosteric small-molecule binding (45, 48, 49, 110), or G-protein and arrestin coupling (111) and guided more rational experimental approaches by suggesting potential interactions or mechanisms, in advance of already available knowledge. These experiments, in turn, were useful for refining or proving model-based predictions.

How can a TSHR model based on already solved crystal structures of other GPCRs be generated? Initial attempts at building TSHR models used those GPCR crystal structure templates available at the time: (1) inactive conformations—rhodopsin [PDB entry 1F88 (112)], beta-2-adrenergic receptor [ADRB2, PDB entry 2RH1 (113), PDB entry 2R4S (114)]; (2) active conformations—opsin [PDB entry 3CAP (115)], opsin in complex with a C-terminal-binding peptide derived from the Gt-protein [PDB entry 3DQB (116)] or active metarhodopsin II (PDB entries 3PXO or 3PQR) (117), the beta-2 adrenergic receptor in complex with agonist and Gs-protein [PDB entry 3SN6 (118)], or the Adenosine-2A receptor in complex with an agonist and a mini-Gs protein [PDB entry 5G53 (119)]. The particular template selection was made based on the specific purpose of the models—like simulation of an inactive versus active conformation [e.g., Ref. (120)] and based on general or local sequence similarities. In the past decade, a large number of new crystal structures from diverse GPCRs were solved, including further aminergic receptors, chemokine, peptidic, or fatty acid receptors [reviewed in Ref. (102) and collected under http://gpcrdb.org/structure (121, 122)]. Consequently, this provokes the question as to what is currently the best structural template to model the SD or the entire structure of TSHR. Based on the overall sequence similarity, the closest single template for modeling the SD of TSHR is the beta-2 adrenergic receptor. However, primary sequence similarity to one single structural template may not be the best option. It is now common to build homology models using not only one template but using several template fragments in order to achieve maximum overlap of individual structural features, e.g., helical kinks or helical length dimensions (103, 123). Actually the TSHR has some of these specific structural properties related to amino acid fingerprints, which are not common in class A GPCRs. They are of high importance for an accurate model, and therefore they are also helpful to estimate the best modeling template. We will therefore extract and describe here a few significant examples important for defining structural properties of the TSHR, and we will also provide an inactive state model that is based on a “multi-fragment” approach (123).

One striking difference between the transmembrane helix (TMH) domain of most other class A GPCRs and the TSHR is that class A GPCRs typically contain a highly conserved proline in position 5×50 [modified Ballesteros and Weinstein nomenclature (124) considering structural alignments of bulges (125)] of TMH5, which is responsible for a bulged TMH5 conformation that causes a kink and twist toward the extracellular end of this helix. However, in the TSHR, there is an alanine (Ala593) in the corresponding position instead of a proline. Based on modeling approaches and mutant studies, in 2011 we suggested that an alanine at position 5×50 in TSHR causes a regular and stable alpha-helical conformation instead of a proline-supported bulge and kink in TMH5 (126). This structural prediction was later confirmed in crystal structures of receptors that do not have a proline at position 5×50 and which do indeed have a regular alpha-helical TMH5 such as the Sphingosine 1-phosphate receptor 1 [alanine in position 5×50; PDB entry 3V2W (127)], the P2Y12 receptor [asparagine in position 5×50; PDB entry 4NTJ (128)], and the lysophosphatidic acid receptor 1 [threonine in position 5×50, LPAR1, PDB entry 4Z34 (129)]. These structural implications for Ala593 in TMH5 of TSHR (126) were recently confirmed by others (104).

Moreover, a methionine (Met637) in TMH6 of TSHR is also a specific feature of this receptor because at the corresponding position (6×48) the majority of class A GPCRs have a highly conserved tryptophan. Replacement of Met637 by a tryptophan led to constitutive activation, indicating a different or altered side chain adjustment at this position in the TSHR (106). Homology models must be built by incorporation of these special functional–structural characteristics, ideally by using structures with the exact match in the respective property. The TMH5–TMH6 arrangement but also that between TMH3 and TMH5 are key features and should be significant for functionalities like the high basal signaling activity of the TSHR (130) or the huge amount of known constitutively activating TSHR mutations (26), whereby these structural features should predestine the TSHR for constitutive activation just by slight amino acid alterations.

To build the most accurate models with implementation of these specific features, a fragment-based modeling approach was developed, whereby templates are selected separately for each TMH and helix 8 using sequence fingerprint motifs and sequence similarity scores (103). The general aim was to select “best-choice” templates based on a logical decision tree or algorithm. This initial idea was transferred into a web server and database [GPCR-Sequence-Structure-Feature-Extractor (SSFE)^1^] to provide the tool to the larger community (123). This initial database contained pre-calculated models for more than 5,000 class A GPCRs (also including different species), but most importantly, this tool generates homology models and structural predictions for sequences of interest uploaded by the user. This method has recently been updated to include all 27 currently available inactive class A GPCR crystal structures for template selection and homology modeling.^2^

**Figure 4:**
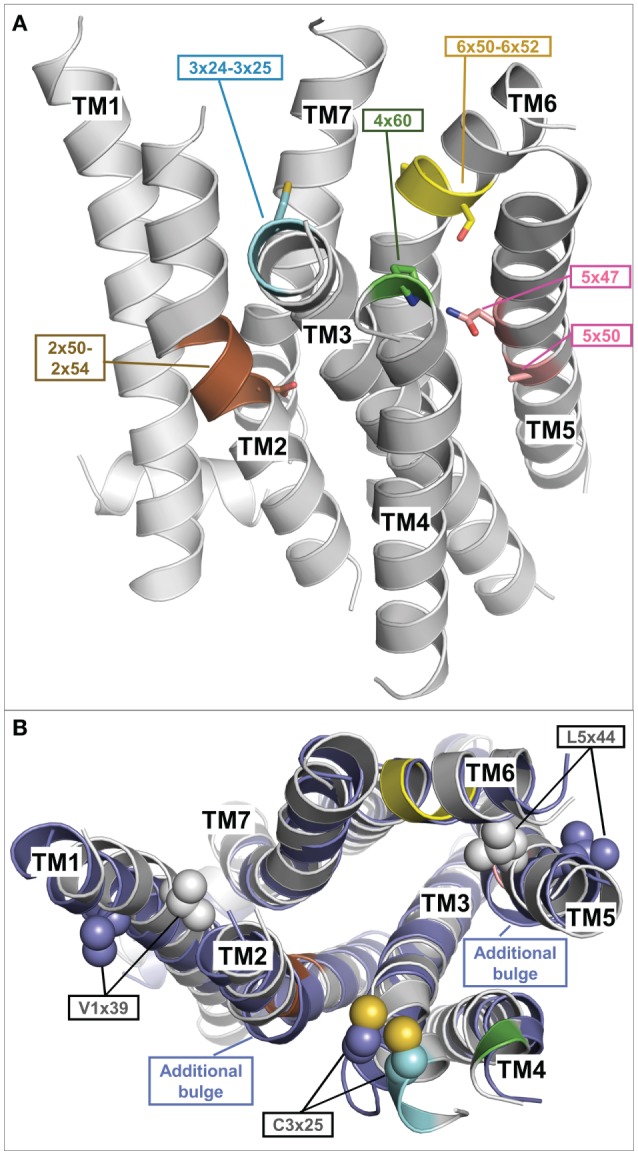
**Fragment-based thyroid-stimulating hormone receptor (TSHR) serpentine domain (SD) model with specific structural features**. **(A)** This TSHR SD model was built from multiple transmembrane helix templates (see Table 1). The best matching fingerprint motifs between TSHR sequence and the selected template transmembrane helix (TMH) fragment are highlighted and indicate a central kink motif for TMH2 (brown: DXXXG at position 2×50 to 2×54), an extracellular kink for TMH3 (cyan: Gly–Cys at position 3×24 to 3×25), an extracellular proline for TMH4 (green: P at position 4×60), a regular central alpha helix for TMH5 (rose A5×50, N5×47), and a strong kink for TMH6 (yellow modified FXXCWP motif at position 6×44 to 6×50, PXS at position 6×50 to 6×52). The remaining TMH templates were selected based on having the highest sequence similarity. **(B)** Comparison of the multiple-template fragment-based model (gray) with the best matching single template TSHR SD model based on the beta-2 adrenergic receptor (PDB entry 2RH1) (blue), which differs in additional bulges in TMH2 and 5 but also in orientations of the side chains V421 (position 1×39) and L587 (position 5×44). Constitutively activating mutations of both residues (104) are rationalized by the fragment-based model when these side chains point toward neighboring helices (gray), but are incompatible with them being orientated toward the membrane as observed in the single template TSHR model (blue).

The inactive TMH model of TSHR generated during this recent update selected 6 of the 27 different template structures for model building (Table 1). Selecting transmembrane helices from different structural templates has the advantage that sequence differences causing slight backbone changes such as bulges or kinks are considered in more detail. Thus compared to using a single template, the multiple fragment approach can achieve an improved accuracy in the predicted models, which is essential for docking of small molecules or virtual screening. The reasons and fingerprint motifs for selecting particular TMH templates for the multiple fragment TSHR model are given in Table 1. For example, the conformation of TMH2 is based on TMH2 from ACM4 receptor (PDB entry 5DSG) since it contains (like TSHR) the fingerprint motif DXXXG at positions 2×50 to 2×54 and has the highest sequence similarity of similarly scoring templates. TMH3 of TSHR is based on TMH3 of AA2AR (PDB entry 4EIY) because of the matching fingerprint Gly–Cys at positions 3×24 and 3×25. TMH5 is based on TMH5 of LPAR1 (PDB entry 4Z34), since like TSHR, there is not only no proline in position 5×50 but also no Phe in position 5×47 and an Asn at that position instead. Three different templates OX1R (PDB entry 4ZJ8), OX2R (PDB entry 4S0V), and P2Y12 (PDB entry 4NTJ) score most highly for TMH6 and are suggested for modeling this helix. We selected the model using human orexin receptor type 1 (OX1R_HUMAN) for further analysis due to it having the highest number of motifs matched and having the best resolution for the X-ray structure. Thus, the resulting TSHR model contains distinct kinks in TMHs 2 and 6 and a straight TMH5 due to the matched fingerprint motifs in these helices (Figure 4A).

**Table 1:** **Template fragments from different G-protein-coupled receptor crystal structures used for building an inactive homology model of the serpentine domain of thyroid-stimulating hormone receptor**.

Helix	Sequence similarity (%)	Suggested transmembrane helix (TMH) fragment template (UniProt entry name—PDB code)	Reasons for template selection (fingerprints)
TMH1	60	ACM2_HUMAN—3UON	Highest sequence similarity
TMH2	57	ACM4_HUMAN—5DSG	DXXXG at position 2×50 to 2×54, highest sequence similarity
TMH3	53	AA2AR_HUMAN—4EIY	GC at position 3×24 to 3×25
TMH4	50	OPSD_TODPA—2Z73	P at position 4×60, highest sequence similarity
TMH5	52	LPAR1_HUMAN—4Z34	No P at position 5×50, no F at position 5×47, N at position 5×47, highest sequence similarity
TMH6	47	OX1R_HUMAN—4ZJ8; OX2R_HUMAN—4S0V; P2Y12_HUMAN—4NTJ	No FXXCWXP motif at position 6×44 to 6×50, PXS at position 6×50 to 6×52, highest sequence similarity; no FXXCWXP motif at position 6×44 to 6×50, PXS at position 6×50 to 6×52, highest sequence similarity; no FXXCWXP motif at position 6×44 to 6×50, highest sequence similarity
TMH7	50	OPSD_TODPA—2Z73	Highest sequence similarity
H8	55	AA2AR_HUMAN—4EIY	Highest sequence similarity

*Reasons and fingerprint sequence motifs for selecting a particular TMH template are given*.

Figure 4B shows a comparison between this multiple fragment model with the best matching single template TSHR model based on the ADRB2 [PDB entry 2RH1 (114)]. The single template model differs not only by additional bulges in TMH2 and 5 and in the orientation of the highly conserved cysteine in TMH3 but also in orientations of the side chains Val421 (position 1×39) and Leu587 (position 5×44) (Figure 4B). Conservative mutations at these positions to isoleucine and valine, respectively, cause constitutive activation (104) and is thus incompatible with them being orientated toward the membrane as observed in the single template TSHR model (Figure 4B). However, the activating roles of these mutations are rationalized by the structural data when these side chains point toward neighboring helices (and thus potential interaction partners), as is observed in the multiple fragment TSHR model (colored in gray in Figure 4B). This clearly demonstrates the advantage of the multiple fragment approach in achieving an improved accuracy in the predicted SD models. Along these lines, recently 16 inactive crystal structures were used to generate multiple-template SD models of the TSHR utilizing another strategy (131). In their approach, Modeller (132) was used to build an averaged model of the TSHR SD by automatically combining all templates.

This also includes the intra- and extracellular loops. For adjusting the extracellular loops of TSHR models, different approaches have been used. SSFE integrated Superlooper2 (133), while others used Monte Carlo refinements (134) and Rosetta protocols (135) for TSHR loop modeling.

The SD model in an active state TSHR conformation can be built on the helix arrangement as observed in the crystal structures of opsin (116), metarhodopsin II (117), adenosine 2A receptor (119), or the beta-2 adrenergic receptor (118), where a huge outward tilt movement of ~8–14 Å of TMH6 were observed compared to the inactive state conformation [e.g., reviewed in Ref. (136, 137)]. The beta-2 adrenergic receptor crystal structure complexed with agonist and Gs-protein (PDB entry 3P0G) served as a template to build the TSHR active state SD model. However, additional TSHR-relevant fingerprints of TMH conformations (described above) were considered while modeling for TMH2 (kink but no bulge) and TMH5 (straight helix).

### TSHR-Interacting Proteins—Hormones, Antibodies, G-Proteins, and Arrestin

The TSHR is a hub for signal transduction between different cellular regions and transduces information from signal inducers (extracellular) toward intracellular signaling molecules. Taking the high number of different GPCRs and ligands into consideration [more than 800 in humans (7, 138)], these ligand/GPCR(s)/effector systems are generally of high evolutionary success and importance (139). The physiological differentiation between particular GPCRs, their ligands, and resulting signaling in one cell or tissue are determined by time occurrence, cell-specific expression levels, ligand/receptor selectivity, and spatial separation, which also holds true for the TSHR under physiological conditions. In addition, for TSHR-interacting proteins like the Gs-protein (140–142) or TSH (143, 144) pathogenic mutants are known. These facts, as well as in context to its interacting proteins makes it very interesting to study and describe the TSHR or to search for further potential interaction partners that are unknown so far. But what is currently known about TSHR-interacting proteins in bound or unbound conformations?

**Figure 5:**
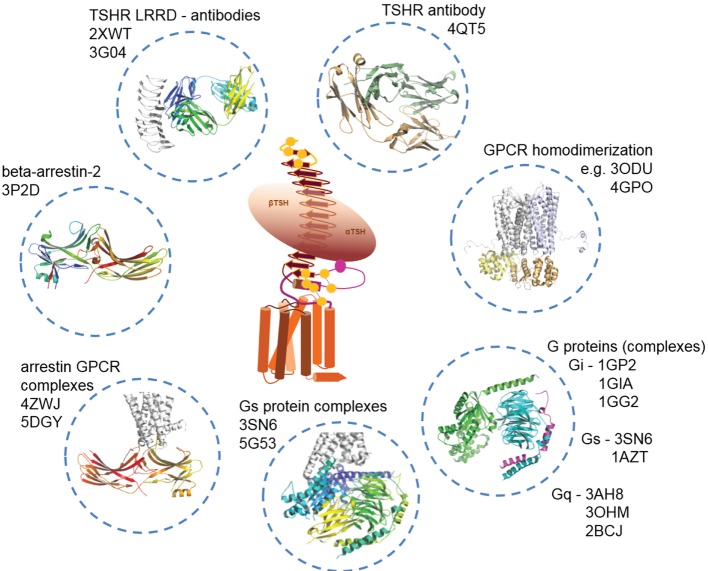
**Available structural information for thyroid-stimulating hormone receptor (TSHR) interaction partners**. As shown in Figure 2, structural information on the TSHR is still limited. However, several interaction partners like autoantibodies (TSH is not solved so far), or Gi, Gs, and arrestin in bound and unbound conformations already have determined structures available. This knowledge can be used to construct larger model complexes as presented in Figures 6, 8 and 10.

In Figure 5, we provide an overview of known TSHR interaction partners and respective available structural information. In brief, TSHR can interact extracellularly with:

i. TSH and thyrostimulin, but no direct structural information is yet available, only structural homology models can be designed based on similarity to existing crystal structures of FSH [PDB entries 1FL7 (145)—unbound state, 1XWD (64) and 4AY9 (65)—bound state] or CG [all structures are in unbound state, PDB entries 1HCN (146), 1HRP (147), 1QFW (148)] (see Figure 2).
ii. Blocking [PDB entry 2XWT (57)] or activating antibodies [PDB entry 3G04 (56)], direct structural information is available in bound conformations, and also the unbound structure of an (inverse agonistic) antibody is available [PDB entry 4QT5 (149)].

In the transmembrane region TSHR can constitute:

iii. Homodimers (150, 151), which can be modeled by using several different GPCR dimer structures (see also Structural–Functional Aspects of TSHR Oligomerization), like from the μ-opioid-receptor [MOR (152)], κ-opioid receptor [KOR (153)], opsin (115), chemokine receptor CXCR4 (154), or the β-adrenergic receptor 1 [β-1AR (155)]. So far, it is unknown whether TSHR also constitutes functionally relevant heterodimers with other GPCRs, but it would be of enormous importance to clarify this question because heterodimerization could have dramatic consequences on TSHR functionalities as known from other GPCRs (156–160) and many different GPCRs are expressed in the same tissues as TSHR [e.g., searchable in Ref. (161)].

Intracellular interaction partners are:

iv. Arrestin, where bound complexes with opsin or rhodopsin are available [rhodopsin/arrestin PDB entries 4ZWJ (162), 5DGY (163)], and opsin/arrestin fragment [PDB entry 4PXF (164)], but also unbound arrestin structures were already determined [e.g., inactive state—PDB entry 3P2D (165)], or pre-active states [PDB entries 4J2Q and 4JQI (166, 167)].
v. Numerous crystal structures of unbound (inactive) G-protein subtypes have been solved, like for Gi [PDB entries 1GIA (168), 1GG2 (169)], Gs [PDB entry 1AZT (170)], and Gq [PDB entries 3AH8 (171), 3OHM (172), 2BCJ (173)]. Based on the beta-2 adrenergic receptor/Gs complex, a bound Gs conformation is also available [PDB entry 3SN6 (118)].

Thus, based on the above as well as the information from Figure 2, it is clear that a specific set of structural information is already available for TSHR and interacting proteins, intracellular and extracellular. Consequently, the available data enables two objectives:

1. The assembling between TSHR and interacting proteins as models of complexes.
2. The estimation of structural transitions between the unbound and bound states for TSHR as well as for the interacting partners.

However, it must also be concluded that much structural information is still missing, such as from the TSHR-binding hormones [TSH, thyrostimulin (174–176)], or TSHR structures themselves, or with bound allosteric ligands or intracellularly complexed partners. Moreover, combined with the missing information of the entire TSHR SD region or the full-length receptor with spatially adjusted domains, the molecular interpretation of functional data from mutagenesis studies or pathogenic findings is an approximation rather than a definitive answer so far. However, in the following section, we describe examples of feasible complex models, which are based on above described structures or homology models.

### Feasible TSHR and TSHR Complex Models

**Figure 6:**
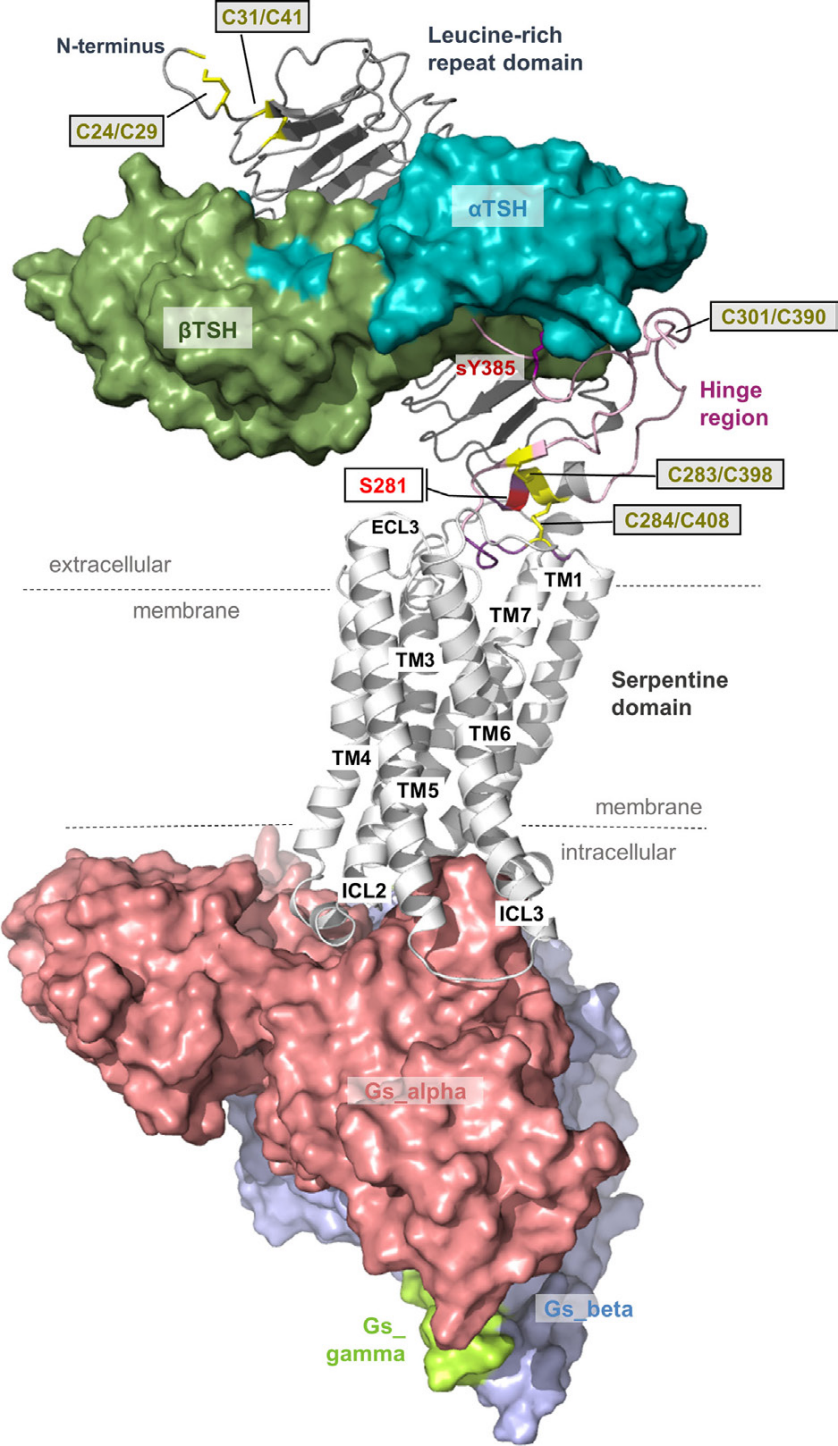
**A thyroid-stimulating hormone receptor (TSHR)/Gs complex model**. The nearly completed complex model between TSHR–TSH and Gs in an active conformation can be assembled based on information summarized in Figures 2 and 3. TSH (or thyrostimulin) binds at two sites in the TSHR, called binding site I (LRRD) and binding site II (hinge region), of which several specific amino acids mediate the contact and specificity for the hormone. This model provides structural information according to the general TSHR scheme in Figure 1, including the detailed disulfide bridges at the extracellular part, localization of the hinge region, or justification of the Gs molecule at the active TSHR structure conformation [based on the beta-2 adrenergic receptor/Gs complex PDB entry 3SN6 (118)].

**Figure 7:**
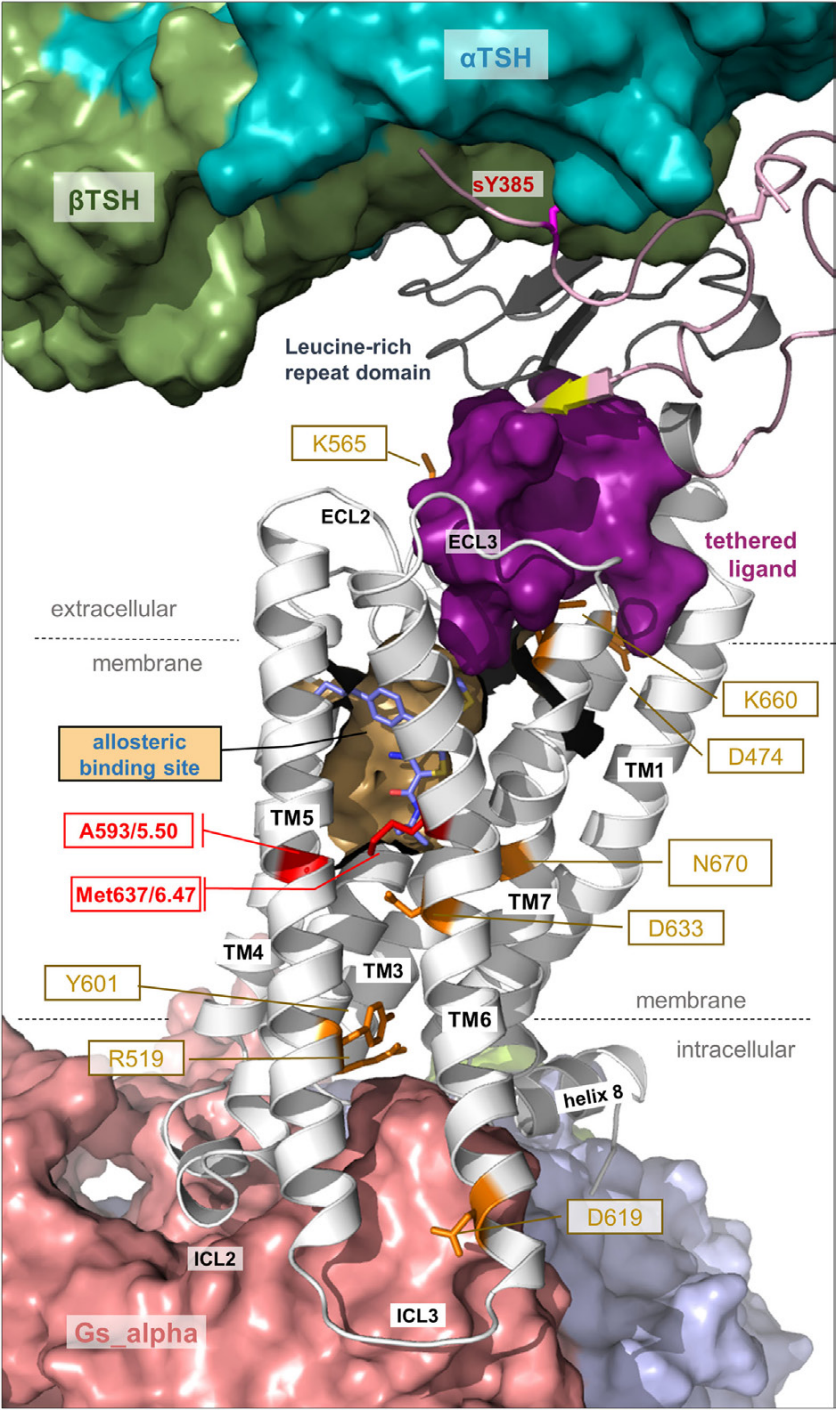
**Details of thyroid-stimulating hormone receptor (TSHR) structure and activation**. This complex model visualizes important determinants and aspects of the TSHR activation mechanism. The hinge region links the LRRD with the serpentine domain and both parts harbor determinants for hormone binding. Ligand-binding triggers conformational changes at a convergent center between the LRRD and hinge region, thereby an inhibitory impact of the extracellular part on the receptor gets abrogated and an “intramolecular agonistic unit” or “tethered internal agonist” close to the transmembrane domain 1 becomes activated (violet surface). This extracellular signal induction is conveyed *via* structural rearrangements of the transmembrane-spanning helices toward the intracellular side. Several amino acids of high structural–functional relevance are involved in receptor activation (orange sticks) by maintaining specific activity-related conformations. They are localized at distinct spatial regions inside the TSHR, and they are interrelated with each other. The resulting active receptor conformation opens a spatial crevice for binding of intracellular interaction partners (Figures 6 and 8). Notably, the TSHR is characterized by specificities in the structural details such as a regular conformation of TMH5 compared to most other G-protein-coupled receptors (GPCRs), having an alanine instead of a proline at the 5×50 position, respectively. Moreover, the TSHR like all other glycoprotein hormone receptors (GPHRs) has a methionine at position 6×47 in TMH6, where usually a tryptophan is located in most class A GPCRs. In addition, it has been shown several times (48, 110, 177) that the known allosteric-binding sites for small drug-like molecules acting on GPHRs are located between the transmembrane helices close to the extracellular loops, which is shown here exemplarily by a partial surface-pocket representation and a bound synthetic antagonist.

**Figure 8:**
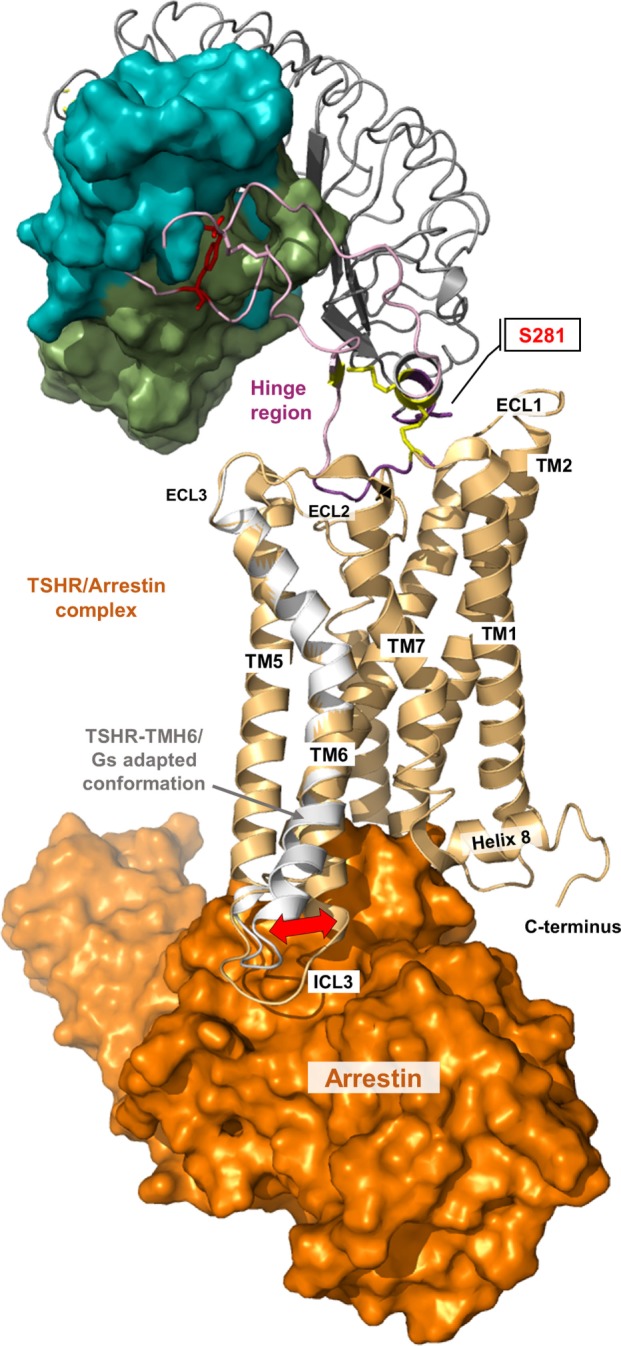
**A thyroid-stimulating hormone receptor (TSHR)/arrestin complex model**. Binding and action of β-arrestin-1 and β-arrestin-2 on TSHR has already been reported (178–181). The putative structural conformation of TSHR adapted to this interacting protein is different to the TSHR/G-protein complex as shown in the presented superimposition of a TSHR/arrestin model (orange surface, complex is based on the crystallized rhodopsin/arrestin complexes PDB entries 4ZWJ, 5DGY) with the TMH6 conformation from the active TSHR/Gs complex (white backbone).

**Figure 9:**
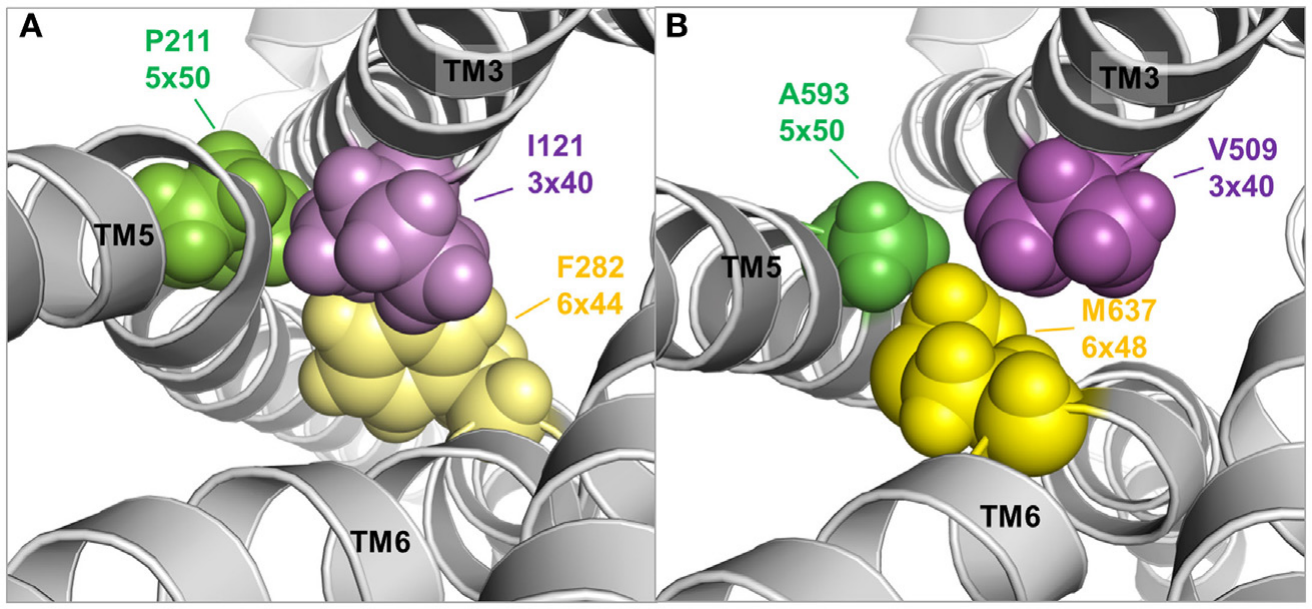
**TMH3–5–6 contact motif in the active state conformations of the beta-2 adrenergic receptor and an active state thyroid-stimulating hormone receptor (TSHR) model**. A specific contact motif between residues in transmembrane helices 3, 5, and 6 is observed in the crystal structure of an active state conformation of the beta-2 adrenergic receptor (118) comprised Ile121 (3×40)—Pro211 (5×50)—Phe282 (6×48) [**(A)**, left panel]. Such hydrophobic contact can also be found in the TSHR model comprised Val509 (3×40)—A593 (5×50)—Met 637 (6×48) [**(B)**, right panel], although the amino acids differ. This contact motif is essential for triggering the active state in the TSHR.

**Figure 10:**
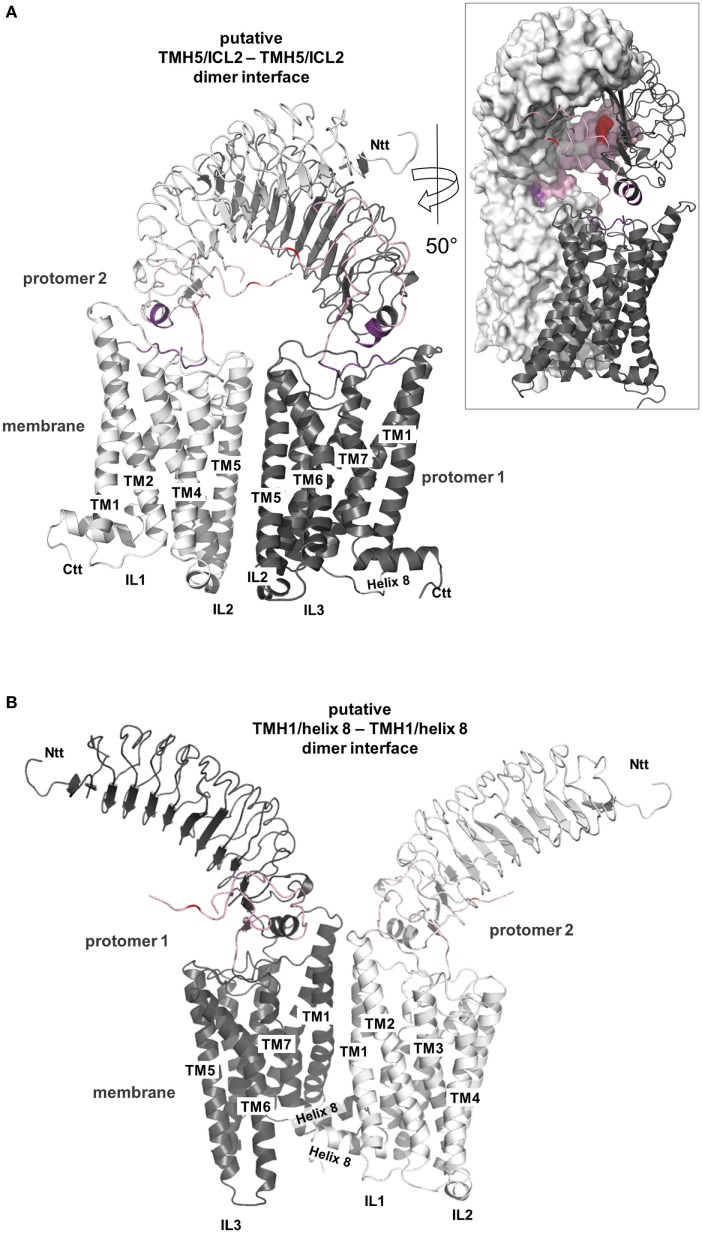
**Putative thyroid-stimulating hormone receptor (TSHR) dimer formations**. A definitive TSHR homodimer interface still awaits experimental evidence but based on the available data it can be summarized that the serpentine domain has the main impact on dimer formation with the extracellular part also contributing (182–184). Already crystallized G-protein-coupled receptor (GPCR) homodimer arrangements are available [reviewed in, e.g., Ref. (185, 186)] and they point to three different potential arrangements between the receptor protomers, at: (I) TMH1–helix 8/TMH1–helix 8, (II) TMH5–TMH6/TMH5–TMH6, and (III) TMH4–ICL2/TMH4–ICL2. These insights can be extrapolated to other GPCR oligomers assuming homology in sequence, structure, and mechanisms and using superimposition here we present two of these putative arrangements for a putative TSHR dimer constellation (150, 151, 187, 188) (entire homology model). In panel **(A)**, a putative TMH5/ICL2–TMH5/ICL2 interface is shown based on the solved dimeric chemokine receptor CXCR4 [PDB entry 3ODU (154)], and in panel **(B)**, a putative arrangement of the protomers with a TMH1/helix 8–TMH1/helix 8 interface is presented based on the opsin-dimer [PDB entry 3CAP (115)]. Both arrangements are feasible and also might occur simultaneously (e.g., in oligomers). In panel **(A)**, the extracellular parts of both protomers get sterically close (see insert with partial surface representation) and hormone binding would need a rearrangement of this extracellular constellation. In panel **(B)**, a symmetric TMH1–helix 8 interface hormone binding would not be influenced by the protomer arrangement.

At the moment, the gap in structural information can only be resolved by building homology models based on the aforementioned crystal structures (Figures 2, 3A,B, 5 and 9). By building individual and complexed homology models, insight into the TSHR SD, the differences between active and inactive structures or between bound and unbound properties of the interacting proteins can be gained. The principal idea of homology modeling is to adapt the already determined homologous structures and respective amino acid sequences (e.g., described in Section “TSHR-Interacting Proteins—Hormones, Antibodies, G-Proteins, and Arrestin”) toward the targets of interest—e.g., TSHR and TSH. This method is appropriate because the structural conservation and similarity of GPCRs is higher than their amino acid sequence similarity (100, 101, 103). We used the structural information documented above (i.–v.) to design the following TSHR-related models in different activity-state conformations:

(1) The hormones TSH and thyrostimulin in bound and unbound conformations based on FSH (free and bound) or CG (unbound) (Figures 2, 6–8).
(2) The full-length TSHR LRRD based on the LRRDs of the TSHR and of FSHR ECD/FSH complexes—as ligand bound conformations (Figure 3).
(3) The LRRD in combination with the hinge region based on the FSHR ECD/FSH complex—active state conformation (Figures 3B and 6).
(4) The partial extracellular TSHR part bound with TSH or thyrostimulin based on the FSHR ECD/FSH complex (Figure 6).
(5) The TSHR extracellular part (LRRD and hinge region) bound with antibodies based on template chimeras between the solved LRRD/antibody complexes and the FSHR/ECD.
(6) TSHR SD in an inactive state (e.g., Figure 4) based on other GPCRs with determined structures.
(7) TSHR SD in active state conformations (e.g., Figures 6, 8 and 9) like from ADRB2 or opsin.
(8) Inactive or active state conformations with bound allosteric ligands (Figure 7).
(9) TSHR SD or full-length TSHR as homomers (in inactive or active states) based on solved dimer structures of other GPCRs like opsin or MOR (Figure 10).
(10) TSHR in complex with arrestin (active state, Figure 8).
(11) TSHR in complex with G-protein (active state, Figure 6).
(12) TSHR homomers in complex with intracellular effectors [assembled active state complex models (Figures 7 and 8) in superimposition with dimeric GPCR crystal structures (Figure 10)].

These models provide insights into the:

- the putative structure and domain composition (Figures 6–10);
- hormone binding-related determinants (Figures 3 and 6);
- determinants of signal transduction at the extracellular region (Figures 3 and 7);
- constitution of the SD in different conformations (Figures 6–9);
- visualizing particular important amino acids for intramolecular signal transduction (Figures 7 and 9);
- TSHR-binding modes with G-protein or arrestin (Figures 6 and 8).

The models outlined above are advanced compared to the few experimentally determined TSHR structures yet they are only approximate models and not necessarily correct or precisely predictive. Functionally supportive data for assembling the SD and the extracellular region are rather rare (135, 189). More detailed methods for building these models are described in our own previous publications on the TSHR or other GPCRs [e.g., Ref. (53, 90, 91, 126, 189, 190)]. However, what can these models tell us or how can they help to visualize mechanisms of the TSHR? In the following sections, we will highlight several important insights related to regulation and action of the TSHR, which are strongly dependent on structural properties.

## Signal Transduction by Structural Reorganization: The TSHR at Work

### Induction of Signaling in the Extracellular Region

Induction of the endogenous signal transduction by the TSHR is triggered extracellularly by TSH (191) or thyrostimulin binding (174–176). The LRRD and the hinge region both harbor determinants for hormone binding [reviewed in Ref. (36, 63)]. Additionally, one specific residue of high importance for TSH binding is a sTyr sTyr385 (76, 92) located in the C-terminal end of the hinge region (Figures 3 and 6). Further amino acids in the hinge region are involved in ligand binding, mainly characterized by negatively charged side chains (53, 192–194). Generally, the hinge region has a drastic influence on hormone binding, structural constitution, and signal transduction, also in concert with the SD [e.g., Ref. (55, 195–201)].

From homology models of the TSHR (91) based on the crystal structure of the extracellular domain of FSHR (65), it was suggested that upon hormone or activating antibody binding a spatial displacement triggers conformational changes at a convergent center between the helical C-terminal end of the LRRD (pivotal helix) and the N- and C-terminus of the hinge region (Figures 3 and 7). The hinge region flexibility agrees with later suggestions that interactions between negatively charged residues in the hinge region and positively charged residues in the LRRD of TSHR are released upon hormone activation (202), or with suggested charged–charged interactions between the LRRD (Glu251) and hinge region (55). From the same models in 2012 (91), it also became clear that serine 281 is located within the short helix at the junction between the LRRD and hinge region (Figure 6). From naturally occurring mutations and mutagenesis studies, this residue is known to be functionally significant (constitutive receptor activation) (69, 70, 72, 73). This serine has also been suggested to interact with the extracellular loop 1 (73), which was recently supported by cross-linking studies (135).

Notably, the hinge region has an inhibitory function on receptor activity as revealed by previous mutational studies (201, 203–205). In addition, it was shown in 2002 that the extracellular N-terminal TSHR part switches from a tethered inverse agonist to an internal agonist (173), although the precise determinants of both (eventually separated) functional units are still not clarified in their entirety because of a lack of experimental structural data. However, in 2004, it was found that the internal agonist comprises specific amino acids (Asp403–Asn406) in the C-terminal hinge region (189) and further experiments refined these insights on the intramolecular agonist unit (196, 200). A recent study with a peptide including Asp403–Asn406 showed that it can act agonistically (90), providing evidence that the internal agonist (assumed for all three GPHR subtypes) is located extracellularly close to TMH1 (90, 189, 201). In conclusion, the TSHR is characterized by a tethered ligand, which is not common in class A GPCRs, but has been described as a mechanism in several particular cases (206). Moreover, the internal agonist is very likely embedded in-between the extracellular loops of the SD (90, 189, 201) and conveys the signal from the extracellular region toward the transmembrane domain (Figure 7). In this regard, it has been shown previously that the extracellular loops trigger the signal cooperatively (207).

### Signal Transport across the Transmembrane Domain

Signal transduction by GPCRs is regulated by a specific rearrangement of particular helices to each other. But how does this process occur at the protein level and how is it regulated in the TSHR? Due to the lack of determined entire structures of the TSHR (and other GPHRs), the question arises how exactly does the extracellularly provided signal gives rise to helical movements. Generally, highly conserved amino acids in the class A GPCRs that are also found in the TSHR contribute to the maintenance of individual activity states and associated conformations by forming specific interactions. These interactions must be modified to facilitate helix movements and for new ones to occur after initial events to stabilize the active state conformation—in interplay with the ligand and the intracellular effector (208–211). It is known that the largest spatial movement related to GPCR activation affects TMH6 around a pivotal helix-kink at the highly conserved proline 6×50 (116, 118). This key event must also be assumed to occur in the TSHR, which is supported by the fact that a huge number of constitutively activating mutants, particularly on TMH6, are known for the TSHR (26, 43).

Moreover, both above described TSHR specificities—the regular alpha-helical conformation of TMH5 and the tightly packed methionine 637 in TMH6—have impact on the hydrophobic helix–helix interfaces between TMH3–TMH5–TMH6, which are important for the transition between the active and inactive state conformation. This is supported by previous studies where a hydrophobic interaction between TMH5 and TMH3 of the TSHR was analyzed by a complementary double mutant Val509Ala/Ala593Val (Val509, TMH3, 3×40; Ala593, TMH5, 5×50) (212). This double mutant led to a functional rescue of the respective single-mutant dysfunctions and provided evidence for a direct hydrophobic interaction of these TMH3 and TMH5 residues. This finding is strongly supported by crystal structures of other GPCRs in the inactive and active state conformation, where an inward movement of proline (in the corresponding position 5×50) toward TMH3 and 6 is observed for the active state such as for the beta-2 adrenergic receptor [ADRB2 (118)] or mu-opioid receptor [MOR (213)], thereby three hydrophobic residues of the ADRB2 located (i) on TMH5 (Pro211, 5×50), (ii) on TMH3 (Ile121, 3×40), and (iii) on TMH6 (Phe282, 6×44) interact tightly as a hydrophobic patch and contribute to the network of interactions that stabilize the active state conformation (Figure 9A). This spatial arrangement of the three hydrophobic residues was termed “PIF motif” or “contact motif” (210). Agonist binding induces these tightly packed hydrophobic interactions resulting in a rotation of TMH6, with a consequent outward tilt movement of the cytoplasmic helical end (Figure 8). Although the corresponding positions differ in sequence in the TSHR, a hydrophobic contact motif is also formed here by the aforementioned Ala593 (TMH5, 5×50) together with Val509 (TMH3, 3×40) and Met637 (TMH6, 6×48), which are subsequently also involved in the conformational active/inactive state transition (Figure 9B). This corresponds with constitutively activating mutations (CAMs) that were already identified at these TSHR positions [Ala593Asn (214), Val509Ala (212), and Met637Trp (106)].

But how are these modifications in the transmembrane region initiated or enabled? What we know is that the extracellular loops connect the helices (Figure 1) and it can be assumed that interactions occur between the TSHR hinge region and the extracellular loops (73, 201). They likely trigger the signal cooperatively toward the transmembrane region (207). In addition, specific loops or parts may also interact with the extracellular ends of certain helices as shown for the ECL2 and TMH6 in the TSHR (105). In conclusion, modifications of the loops can be transferred directly to interacting or connected helices, which are in line with reports in other GPCRs, where a salt bridge facilitates a link between the loops and receptor activation (215).

Second, signal transduction in the TSHR is not a single line of information flow but rather a multitude of synchronized sequences of events occurring. This assumption is made based on the fact that several previously reported inactivating or activating mutants at distinct amino acid positions are located at different receptor regions (Figure 7). Well investigated and significant examples are Lys660 in the TMH6/ECL3 transition (216), Lys565 in the ECL2 (105), Asp474 in TMH2 (217), or Glu409 in the transition between the hinge region and TMH1 (90) (Figure 7). Furthermore, Asp633 (TMH6) and Asn670 (TMH7) (107, 218, 219) are located in the central part of the domain core; and Tyr601 (220) or Asp619 (221, 222) is in the transmembrane region close to the intracellular site. In consequence and in contrast to the predominantly hydrophobic interfaces between TMH3–TMH5–TMH6, the helix–helix interfaces between TMH3, TMH2, TMH6, and TMH7 are characterized by the occurrence of essential hydrophilic contacts, e.g., at the highly conserved positions Asp2×50 or Asn7×50 (107, 219).

These hydrophilic contacts are complimented by conserved water molecules localized close to the mentioned conserved residues (103). Together, they constitute a network of intramolecular and water-mediated interactions (223) that are important for stabilizing GPCR structures by linking TMHs (224–226). Molecular dynamic simulations of class A GPCRs suggested an intrinsic water pathway, interrupted in the inactive state by hydrophobic layers of amino acid side chains, which change their conformation upon agonist binding leading to a continuous water channel. It is suggested that Tyr7×53 of the NPXXY motif is of importance in this context (227). Receptor activation probably leads to a rearrangement and an extension of the water network [for example, Ref. (90, 107)] from the ligand-binding site to the cytoplasmic surface (228, 229), at least for specific GPCRs. As well as water, allosteric sodium has also been observed in antagonist/inverse agonist bound class A GPCR structures in a highly specific arrangement between TMH2 and TMH7 (224). During activation, the sodium pocket collapses and the ion translocate toward the cytoplasm. However, it seems that not all GPCRs possess this pocket, such as visual opsins which instead have ordered water molecules between Asp2×50 and Tyr7×53 [PDB entry—4X1H (228)]. These observations underline the integral role of water molecules in GPCRs.

Apart from extracellular activation by its endogenous hormone ligands and autoantibodies, the TSHR signaling can be modulated by small-molecule ligands (SMLs) (52). Investigation of a potential allosteric-binding pocket for SMLs within the transmembrane domain (Figure 7) by modeling-driven mutagenesis led to the identification of distinct CAMs, including Val421Ile, Tyr466Ala, Thr501Ala, Leu587Val, Met637Cys, Met637Trp, Ser641Ala, Tyr643Phe, Leu645Val, and Tyr667Ala (106), and silencing mutations such as Val424Ile, Leu467Val, Tyr582Ala, Tyr582Phe, Tyr643Ala, and Leu665Val (230). These positions not only indicate key amino acids covering the allosteric-binding pocket of the TSHR but also positions where the TSHR conformation can be changed to an active or inactive state. Mapping these residues onto a structural model of TSHR indicates locations where SML agonists or antagonists enhance or impair signaling activity (231). These signaling sensitive amino acids are also compiled in the web-based resource “SSFA-GPHR” (41–44).^3^

### Intracellular Binding and Activation of Signaling Effectors

All amino acids of the intracellular TSHR loops were already investigated by site-directed mutagenesis studies (111, 221, 232–235). Moreover, pathogenic mutations at these receptor parts were also identified in patients (236–239). These mutants and site-directed studies revealed that the entire set of the three ICLs and helix 8 contribute to induction of intracellular signaling by the TSHR, although differences concerning the impact on specific signaling pathways has been observed. Diverse activation pathways in class A GPCRs converge near the G-protein-coupling region (240). In principle, GPCR-mediated G-protein activation is characterized by structural shifts inside and between the G-protein subunits to each other, followed by exchange of GDP for GTP in the alpha-subunit and (partial) separation of the Gα- from the Gβγ-subunits (241). This opens up interfaces to further contact partners (242). These events at the intracellular effector are induced by binding to the receptor in predisposition (without intracellular effector but with a bound agonist).

The intracellular effector can bind to the TSHR by fulfilling two criteria: (i) a spatial fit and (ii) an interaction surface that does not preclude binding, rather being supportive. So far, it is not known for GPCRs how exactly selectivity for a certain G-protein subtype is determined directly on the receptor. GPCRs with a preference for a particular G-protein subtype like Gs or Gq could not be allocated yet to a specific set of amino acids in the intracellular site. Additionally, receptor selectivity on the intracellular receptor site can be altered by making an amino acid substitution that repulses a specific effector (biased inactivation), and this is indeed the mechanism of several inactivating mutations in the intracellular TSHR loops, where, for instance, Gq activation is abolished but not activation of Gs [e.g., mutation Phe525Lys (243)]. This, in turn, would mean that selectivity is not associated with a complementary interaction pattern, it might be (theoretically) that selectivity in binding should be reached by a specific exclusion of effector subtypes due to small changes in the shape of the promiscuous receptor G proteins binding interface.

What is known concerning binding of intracellular effectors to the TSHR? As noted above, a huge amount of functional data from amino acid substitutions in relation to G-protein activation (not for arrestin binding) is already available and based on these data first molecular models of a putative TSHR/Gq-protein complex were previously generated (111). This can now be extended by incorporation of TSHR/Gs (Figure 6) and TSHR/arrestin (Figure 8) complex models based on recently determined structural complexes of other GPCRs [based on the beta-2 adrenergic receptor/Gs complex—PDB entry 3SN6 (118), or the rhodopsin/arrestin complex—PDB entry 4ZWJ (162)]. The intracellular loop 1 (ICL1) contributes to G-protein binding but the amino acids have a different impact (111). Of particular interest is Arg450 at the transition between ICL1 and TMH2, where several cases of naturally occurring inactivating mutations were reported (244–247). Amino acid Arg450 may directly interact with Gα as suggested by our homology model, e.g., with Gln390 in the C-terminal α5-helix of Gαs (111). However, the middle part of the ICL1 is exclusively oriented toward the beta-subunit of the G-proteins and mutations in this region only decrease inositol phosphate (IP) generation, not cAMP accumulation (Leu440Ala, Thr441Ala, and His443Ala). Of note, it was reported for the MOR that initial interactions between the G-protein and intracellular loop 1 and helix 8 may be involved in G-protein coupling specificity and that TMH5/6 contribute later in the process of complex formation (248). This finding would be in general agreement with our suggestion that ICL1 is also involved in G-protein coupling by the TSHR.

In addition, the intracellular loop 2 (ICL2) is significantly involved in G-protein activation in the TSHR (221, 243). Amino acids Met527, Arg528, and Asp530 are critical for both Gs and Gq activation, whereas alanine mutations of Ile523, Phe525, and Leu529 only impaired Gq-mediated signaling but not the Gs-mediated cAMP accumulation. Alanine mutations of Met527, Asp530, and Arg531 also caused impaired basal cAMP accumulation (120), which indicates involvement in Gs binding also in the basally active state conformation. Moreover, we suggest that the ICL2 conformation is helical (Figure 6) as supported by several crystal structures of diverse GPCRs, specifically in complexes (118, 162). In addition, the transitions between TMH5–ICL3–TMH6 were identified as being important for G-protein activation, whereby single substitutions of Tyr605, Val608, Lys618, Lys621, and Ile622 selectively decrease Gq activation (220, 221). By contrast, mutations at Asp617 and Asp619 cause constitutive receptor activation for the Gs-mediated pathway (218, 221, 239, 249).

Finally, these mutation-based studies at all three ICLs have shown that the binding modes between TSHR and Gs versus Gq do partially overlap, while completely inactivating mutations were only found for the receptor/Gq complex. The fact that Gq-mediated signaling, but not Gs-mediated cAMP accumulation, can be impaired by single side chain substitutions suggests that Gq binding is more fine-tuned than Gs binding. In strong relation to this might be the observed high basal activity for cAMP accumulation by TSHR, which is related to a permanent binding capacity and activation of Gs (130). The differences between Gs and Gq activation must be deciphered in more detail by determination of complex structures.

Moreover, so far, no experimental data from mutagenesis studies or structure determination are available concerning binding of arrestin to the activated TSHR, although arrestin binding is known to be of functional importance, e.g., for physically blocking further G-protein coupling and initiating the receptor shut-off (178–181). Activated GPCRs are phosphorylated by specific kinases on multiple sites at the C-terminus. In the inactive or basal state, arrestins are unable to bind activated TSHR, and interaction with several receptor-attached phosphates is critical for such an interaction. GPCR binding by arrestin is often discussed in terms of two events. Arrestin forms a low-affinity pre-complex with the receptor, in which the phosphorylated receptor C-terminus replaces the C-tail of arrestin and thereby gains access to the high number of basic residues in the N-domain area (166, 167). C-tail displacement induces numerous conformational changes in key motifs and an overall domain rearrangement in arrestin that allow the second and tight-binding event of the activated receptor and the formation of a high-affinity complex. A key interaction of this high-affinity complex is the binding of the so-called finger loop region in arrestin to the intracellular-binding crevice of the activated receptor (162, 164), thereby the finger loop adopts a near helical structure and interacts with the highly conserved *E(D)RY* motif of the activated receptor. Remarkably, arrestin (namely, the near helical finger loop region) and G-protein (namely, the C-terminal alpha5 helix in the Galpha subunit) share a common binding crevice on the activated receptor (164). On the basis of the low-resolution crystal structure of peptide linker-fused rhodopsin–arrestin complex (162), a putative TSHR/arrestin complex model was created (Figure 8). The putative structural conformation particularly in TMH6 and ICL1-3 of TSHR adapted to this interacting arrestin model is slightly different to the TSHR/Gs-protein complex. However, until now, there are still many unanswered and unresolved questions due to the limited structural and biochemical knowledge of arrestin binding to GPHRs.

### Structural–Functional Aspects of TSHR Oligomerization

Constitution of homo- and heteromers has been demonstrated for several members of different GPCR groups (250–253). Oligomerization is a biological tool for fine-tuning signaling and hence also physiological function (254–256), which is also relevant to endocrinology (257) and in pathological conditions (258–262). It is well documented that dimerization or oligomerization can have an impact on signaling properties as well as ligand binding (263, 264), signal transduction (265, 266), or cell-surface expression (267). Thus, oligomerization has been demonstrated to be a common and important feature of GPCRs including TSHR. What is known regarding TSHR oligomerization so far?

- TSHR oligomerization (150, 151, 187, 188) occurs early in the endoplasmatic reticulum and is suggested to be crucial for proper receptor expression (268).
- TSHR probably forms higher order homomers rather than dimers (182) and the extracellular region participates in oligomerization, while the main protomer contact is most likely located at the transmembrane-spanning part (Figure 10) (183).
- A recent study revealed that two TSH molecules bound to a TSHR homodimer are required to activate not only Gs but also Gq (269).
- It has been debated as to whether TSH influences dimer formation (183, 270). On the one hand, it was proposed that oligomeric TSHR rapidly dissociates into active monomers upon TSH binding (271). On the other hand, dimerization was found not to be affected by ligand binding (182).
- Functionally dominant-negative effects have been shown for partially inactivating TSHR mutations (272). TSHR di- or oligomerization presents a molecular explanation as to why these TSHR mutations exhibit a phenotypic effect even in the heterozygous state of an inactivating mutation (273).
- By contrast, CAMs do not influence dimeric TSHR arrangements (182, 274).

One of the basic questions concerns TSHR oligomer organization from the structural perspective. Interfaces (contact-regions) between GPCR protomers were found under experimental conditions for different GPCRs, for instance, at the region of ICL2–TMH4 (275–277), TMH4–TMH5 (278), or TMH5–TMH5 (279–281). Most importantly, several crystal structures of dimeric GPCR complexes were determined, e.g., the μ-opioid-receptor [MOR (152)], κ-opioid receptor [KOR (153)], opsin (115), chemokine receptor CXCR4 (154), and the β-adrenergic receptor 1 [β-1AR (155)]. Dimer interfaces are observed between TMH5–6, e.g., in the crystal structure of the CXCR4, or in the case of opsin, KOR, and β-1AR, the protomer interface is located between TMH1 and helix 8. Due to these repeated findings in the dimeric crystal structures, it can be postulated that class A GPCRs tend to have a preference to form protomer contacts at TMH1, helix 8, TMH5, and the ICL2–TMH4 transition.

Detailed characterization of TSHR oligomerization pointed to the SD as a main determinant for intermolecular receptor–receptor interplay and indicated that the extracellular receptor region might participate in this constellation (183, 184, 282). Recent studies suggested that the TMH1 is a main contact in the SD of the TSHR (283), which is in accordance with several of the crystallized GPCR interfaces reported above [e.g., the KOR dimer interface at TMH1–helix 8; PDB entry 4DJH (153)]. In line with this finding and with the published crystalized dimers, we provide molecular homology models of two putative TSHR dimer arrangements (Figure 10). In a putative symmetric TMH5–TMH5 interface, the TSHR would have additional side chain contacts at the extracellular side between TMH5 and TMH6 (Figure 10A). In a putative contact arrangement between TMH1–helix 8 (Figure 10B), TMH2 would contribute to the protomer contacts. A striking difference between both general orientations of the protomers is the relative orientation of the extracellular parts. Because it is so far unknown how the extracellular N-terminal LRRD and hinge region is arranged relative to the SD, the correct TSHR–TSHR constellation is unknown. According to our current homology models and arrangement of the ECD relative to the SD (Figure 6), a TMH5–TMH5 interface would result in sterical clashes between the extracellular parts and hormone binding would require initial structural modifications. In a TMH1–helix 8/TMH1–helix 8 protomer arrangement, the ECDs of both receptor molecules (models) would be freely accessible for the hormone molecules. In any case, it is reasonable to assume that both transmembrane interfaces occur simultaneously in higher order complexes of the TSHR [as observed for the β-1AR (155)], which is probably functionally relevant for properties such as negative cooperativity in ligand binding caused by lateral intermolecular allosteric effects and/or negative intramolecular cooperative effects (183, 284).

Interestingly, the structure of the FSHR extracellular region with bound FSH was solved as a trimeric complex comprised three individual receptor/ligand units (49), while the previously solved FSHR/FSH complex with a shorter LRRD and without the hinge region (64) is a dimeric LRRD/hormone complex. Furthermore, in these two partial FSHR structures, interactions between the respective protomers are not similar, which might indicate flexibility in the arrangement or artificial constellations based on the crystallographic method. However, the trimeric-structure organization for GPHRs should be kept as one of various options for a multimeric receptor organization, since it also fits to several functional data (86, 285).

## Open Questions and Future Directions

In summary, well-defined structural rearrangements and interaction events between different proteins accompanies and characterizes the TSHR activation process. Any modification such as substitution of interacting amino acids may affect the resulting signaling, which is supported by a huge number of naturally occurring mutations in addition to designed inactivating or activating receptor mutants (41–44). Many insights concerning the TSHR structure in relation to detailed and general functions were already identified. This information is useful for deciphering the mechanisms of signaling or pathogenic conditions at the molecular level. However, we also draw attention to the lack of structural information, meaning that the main open questions concern the entire receptor structure—with and without the “C-peptide,” with interaction partners (arrestin or G-proteins) or the exact oligomer constitution. For instance, the bound TSH structure in complex with TSHR would be hugely beneficial for many TSHR-related studies, including the improved directed development or refinement of medical therapeutics targeting the TSHR. Finally, the dynamic signaling process considering all known (and so far unknown) interaction partners resolved in time and cellular localization [also intracellularly (180, 181, 286–290)] would push the field enormously toward a comprehensive understanding of the TSHR, including suggested extra-thyroidal actions (29, 34, 291–296).

## Author Contributions

All authors have worked together on the manuscript in a back-and-forth procedure providing substantial contributions to the conception and interpretations. All authors have proofread the final version. In detail: GKleinau: conceptual contribution, major contribution to the content, generated homology models and their figures, and management of literature; CW: wrote modeling strategies especially for transmembrane domain modelling paragraphs, generated models and their corresponding figures, table, and checked English language; AK: wrote contributions about bioinformatics information resources and generated figures; HB: revised critical contributions about pathogenic and natural mutations of the TSHR; PM: wrote contributions about ligand binding and interaction; PS: wrote contributions concerning crystal structure interaction, especially concerning G-protein and arrestin interaction; GKrause: concept development, wrote and coordinated writing of the manuscript, and generated model figures.

## Conflict of Interest Statement

The authors declare that the research was conducted in the absence of any commercial or financial relationships that could be construed as a potential conflict of interest. The reviewer, MM, and handling editor declared their shared affiliation, and the handling editor states that the process nevertheless met the standards of a fair and objective review.

## Footnotes

^1^http://www.ssfa-7tmr.de/ssfe.

^2^http://www.ssfa-7tmr.de/ssfe2.

^3^http://www.ssfa-gphr.de.

## Abbreviations

GPHR, glycoprotein hormone receptor; LHCGR, lutropin/choriogonadotropin receptor; FSHR, follicle-stimulating hormone receptor; TSHR, thyroid-stimulating hormone receptor; TSH, thyroid-stimulating hormone; GPCR, G-protein-coupled receptor; TMH, transmembrane helix; ECL1/2/3, extracellular loops 1/2/3; ICLs 1/2/3, intracellular loops 1/2/3; SD, serpentine domain; CAM, constitutively activating mutation; WT, wild type; ECD, ectodomain; IP, inositol phosphate.
